# Performance Analysis of Cost-Effective Miniature Microphone Sound Intensity 2D Probe

**DOI:** 10.3390/s20010271

**Published:** 2020-01-03

**Authors:** Witold Mickiewicz, Michał Raczyński, Arkadiusz Parus

**Affiliations:** 1 Faculty of Electrical Engineering, West Pomeranian University of Technology, 70-310 Szczecin, Poland; michal.raczynski@zut.edu.pl; 2 Faculty of Mechanical Engineering and Mechatronics, West Pomeranian University of Technology, 70-310 Szczecin, Poland; Arkadiusz.Parus@zut.edu.pl

**Keywords:** sound intensity, intensity probe, pp-probe, sound source localization

## Abstract

This article presents the functional properties of modified versions of the 2D pressure–pressure intensity probe allowing us to determine the vector of sound intensity on a plane using a mechatronic system with one or two miniature electret microphones. The introduction contains basic information about the application areas of the sound intensity and its measurement problems. Next, the principle of operation of the probes and the construction of the prototype measurement system are described. It was subjected to comparative analysis for the stability of obtained results and accuracy of directional characteristics in free field conditions. For this purpose, experiments were conducted to analyze the flow of acoustic power in an anechoic chamber using both (one- and two-microphone) probes. The results were used for a comparative metrological analysis of the described methods and to indicate the advantages and disadvantages of both constructions. The next part of the article presents an experiment concerning the measurement of the sound intensity impulse response of a room, which is an example of practical use of the probe to analyze reflections in the room, which can be used in sound engineering and architectural acoustics.

## 1. Introduction

Localization of acoustic energy sources and visualization of the vector distribution of the acoustic field around them have, on the one hand, cognitive applications and, on the other hand, many practical applications. The most important applications are: localization of noise sources, measurement of acoustic power in the presence of interference [1], support for voice communication systems, spatial sound engineering in audiovisual systems [2], acoustic localization of threats in security systems, and many others [3]. For this reason, it is important to develop commonly available acoustic sensors that enable the above-mentioned functionalities to be realized.

The basic element of a spatial perception system is the presence of a sensory system containing at least two pressure sensors at some distance from each other. In the case of human beings, thanks to their two ears, they are able to locate sound sources based on the recording of pressure signals characterized by an interaural time difference and an interaural intensity difference, variable in the function of the angle of attack of a sound wave. This bionic approach to the spatial location of sound sources is widely used to produce spatial sound recordings (various types of two- and multi-microphone systems) [4,5]. Today, we also know more advanced solutions using microphone arrays and digital signal processing algorithms for spatial filtering—beamforming or acoustic holography [6]. However, these methods require an increasing number of pressure transducers and multi-channel acoustic signal acquisition and processing systems and also the large size and complexity of the support constructions. For this reason, the use of such techniques is difficult or impractical in many fields.

An alternative to beamforming (which is mainly based on signal processing of sound pressure) are methods based on the use of vector quantities describing the acoustic field, i.e., the acoustic particle velocity and intensity. The sound intensity measurement technique was developed in the 1930s, but the first successful measurements of this quantity date back to 1970 [7]. The first measurement devices were launched in 1980 and 10 years later the first standard describing the method of measuring the sound intensity was published. In this method, the acoustic particle velocity component is determined by determining the acoustic pressure gradient by synchronous measurement at two points in space and allows the acoustic particle velocity component to be determined in one plane [8,9]. A sensor of this type is a pressure probe type p–p (pressure–pressure). In order to determine the spatial distribution of the sound intensity, it is necessary to use three pairs of mutually perpendicular microphones (there are also known solutions using only four microphones for this purpose). Unlike sound pressure measurements (scalar quantity), sound intensity measurements are not trivial. It requires specialized equipment and precision of electroacoustic transducers [10,11]. These facts influence the high price of these types of solutions. There are also limitations of determining the sound intensity over a wide frequency range, related to the physical size of the intensity probe.

With the development of miniaturization, there has been a breakthrough in the measurement of acoustic particle velocity and sound intensity. At the beginning of the 21st century, new sensors were introduced to the market, based on a different principle, known as Microflown [12]. It is a p–u-type sensor (pressure–velocity), which means that the component of the acoustic particle velocity is determined using a different physical phenomenon than the occurrence of the acoustic pressure gradient. It is an anemometric sensor with hot wires. In this sensor, the movement of acoustic particles causes heat to flow between areas around two conductors placed close to each other and creates a temperature difference between them. A change in temperature causes a change in resistance that can be measured with an electronic system [13]. This sensor has introduced a new quality of sound intensity measurement, mainly due to its small dimensions and the ability to determine sound intensity over a wide frequency range. Unfortunately, this sensor also has disadvantages. Due to the principle of operation, its failure-free operation time is relatively short (about two to three years of intensive use) in relation to the high price.

There is also a recurring interest in the above-mentioned method of measuring sound intensity using the p–p probe. A major difference to previous solutions based on the use of expensive, precisely adjusted pairs of microphones is the use of miniature electret microphones (commonly used in audiological equipment) or MEMS (microelectromechanical system) microphones widely used in mobile phones. Exact microphone selection is now replaced by procedures for the correction of amplitude and phase-frequency characteristics. The correction is based on real parameters of used microphones obtained from direct measurement. A final conformity of transducers (removing the discrepancies between them) is obtained by digital filtering. This approach is presented, for example, in the works [14,15,16]. Like any measurement method, despite its many advantages, it also has some disadvantages. The main problem is, first of all, the need for preliminary calibration and estimation of correction coefficients. The proposed procedures require the use of anechoic chambers and specialized equipment. Furthermore, due to the long-term change in the parameters of individual microphones in the probe, it is expected that the necessary calibration process must be repeated periodically to be sure of the metrological parameters of the probe.

In the opinion of the authors, an important aspect that influences a stagnation in the wider use of intensity methods is the cost of equipment, especially professional intensity probes. The aim of the research presented in this paper is an objective evaluation of the metrological quality of modified intensity probes based on the principle of operation of p–p probes, made with the use of fewer inexpensive transducers and a mechatronic positioning system.

## 2. Principle of Operation of the Intensity Probe

An acoustic field is most often described by two physical quantities: sound pressure and acoustic particle velocity. Another quantity, sound intensity, is the function of these two quantities. Due to the simplicity of the measurement procedure, the sound pressure *p_a_* is most often measured, which is a scalar indicating the instantaneous change in pressure around a constant value (in the case of air, it is usually the atmospheric pressure):

(1) $$p_{a}=p\left(t\right)-p_{0}$$

where $\;\;p_{0}$ is the atmospheric pressure, p(t) is the instantaneous value of pressure, and t is a time. Equation (1) describes the instantaneous value of the sound pressure, which can be both positive and negative and varying around the constant value of atmospheric pressure. The meters used in acoustical measurements always determine the positive Root Mean Square (RMS) of the sound pressure according to the formula:

(2) $$p_{aRMS}\left(t\right)=\sqrt{\frac{1}{T}\overset{t}{\underset{-\infty }{\int }}p_{a}^{2}\left(\tau \;\right)e^{-\frac{t-\tau }{T}}d\tau }$$

where *T*-value is the detector’s time constant (in sound level meters, values of 125 ms (FAST mode) and 1 s (SLOW mode) are most frequently used) and τ is the independent variable (time).

The scale of RMS values heard by a human ear is very wide: 2 × 10^−5^ Pa to 200 Pa. For this reason, it is comfortable to use decibel measurement and to determine the sound pressure level (SPL):

(3) $$SPL=20\log\left(\frac{p_{aRMS}}{2\cdot 10^{-5}\mathrm{Pa}\;}\right)$$

Thus, defined scalar quantities measured in a single point of space do not carry any information about the direction (sense and orientation) of acoustic energy propagation.

The propagation of the acoustic wave causes local movement of the medium in which the wave propagates. It is the movement of the acoustic particles. An acoustic particle is a correspondingly small volume of a medium of a size much smaller than the wavelength. At the same time, it contains so many molecules of the medium that it can be assigned the whole volume averaged thermodynamic parameters (e.g., pressure). Acoustic particles can be assigned an acoustic particle velocity. In contrast to acoustic pressure, it is a vector and has three components in the Cartesian coordinate system:

(4) $$\vec{u}\left(t\right)=\vec{i\;}u_{x}+\vec{j\;}u_{y}+\vec{k\;}u_{z}$$

where *i*, *j*, and *k* are unity vectors of coordinate system.

Using the definition of acoustic particle velocity, it is possible to introduce the concept of sound intensity (vector quantity), which describes the flow of acoustic energy in a medium. The instantaneous value of the sound intensity *I_inst_(t)* can be expressed as the product of the sound pressure *p_a_(t)* and the velocity of the sound particle $\vec{u}\left(t\right)$:

(5) $$\vec{I_{inst}}\left(t\right)=p_{a}\left(t\right)\cdot \vec{u}\left(t\right)$$

The mean value of the instantaneous sound intensity is called the active component of the sound intensity and is determined as follows:

(6) $$\vec{I_{act}}=\frac{1}{T}\int_{0}^{T}\vec{I_{inst}}\left(t\right)dt$$

The unit of sound intensity is $\frac{W}{m^{2}}\;$.

The instantaneous sound intensity is a vector quantity with its orientation consistent with the vector of acoustic particle velocity. The magnitude and direction of this vector, however, depends on the sound pressure value. Since, as mentioned earlier, the instantaneous sound pressure values can be positive or negative, the intensity vector direction can be the same as the velocity vector or the opposite. This means that the sound intensity is influenced by the phase relation between the pressure wave system and the acoustic particle velocity wave system. In the free far-field, where the observed waves can be considered as flat, the phase shift between the pressure wave and the velocity wave is zero, the direction of the sound intensity vector coincides with the propagation direction, and the direction indicates the energy flow from the source to the surrounding space.

The observation of the intensity of sound thus gives a more accurate view of the phenomena in the acoustic field, taking into account the interactions of many sources (real and apparent) and their individual amplitude-phase characteristics. Hence, the authors’ interest are in the problem of intensity measurements and their use in sound engineering (especially in the auralization of recordings) and architectural acoustics—the relationship between acoustic properties of rooms and time–space analysis of intensity impulse responses of rooms.

Nowadays, two types of intensity probes are the most common: the p–u probe and the p–p probe. In the p–u probe, pressure and acoustic particle velocity are measured with the use of different types of sensors. A microphone is used to measure the pressure and an ultrasonic (Norsonic, Tranby, Norway) or hot-wire anemometric transducer (Microflown, Arnhem, The Netherlands) is used to measure the acoustic particle velocity. In the p–p probe, the acoustic particle velocity is determined indirectly from the pressure gradient measured by a pair of microphones placed in close distance to each other according to the Euler’s linearized equation:

(7) $$\vec{u}\left(t\right)=-\frac{1}{\rho }\int^{\;}\frac{\partial p\left(t\right)}{\partial \vec{x}}dt$$

where *u*—acoustic particle velocity; *ρ*—density of environment; *p*—acoustic pressure; *x*—space variable.

After discretization, Equation (7) takes the following form:

(8) $$\vec{u}\left(t\right)=-\frac{1}{\rho }\int_{-\infty }^{t}\frac{p_{a1}\left(\tau \right)-p_{a2}\left(\tau \right)}{\Delta x}d\tau$$

The gradient of acoustic pressure is substituted by the finite difference of two acoustic measurements done by a pair of microphones, which should be identical. The lower integration limit is the beginning of the test signal. To measure one, two, or three components of the sound intensity vector accordingly one, two, or three pairs of microphones are necessary, placed orthogonally to each other. The upper measurement frequency range is limited by the proper approximation of a derivative by finite difference. The low frequency range is limited by phase mismatch error and noise of the measured signal. The typical distance is 12 mm and provides measurements in the frequency range: 125 Hz–5 kHz. A general disadvantage of p–p probes is the necessity to use microphones that have identical amplitude and phase-frequency characteristics. In practice, it is impossible to obtain perfect matching. Thus the complex calibration procedures and/or correction of characteristics by analog or digital filters are necessary.

## 3. Modification of the Pressure–Pressure Method

In the case of measurement of acoustic systems whose parameters can be assumed to be constant during the measurement procedure and where the measurement can be performed repeatedly by emitting identical excitation signals, the design of a standard p–p probe can be modified. Firstly, all the components of the intensity vector can be measured using a two-microphone probe type p–p, positioning only one pair of microphones accordingly. Further on, each component can be determined based on a sequence of measurements with one microphone in successive positions corresponding to the distribution of microphones in classical p–p 1D, 2D, or 3D probes. Such an approach was already mentioned many years ago [7,8], but only nowadays it is practically feasible thanks to the development of computer measurement systems guaranteeing proper synchronization of the generation of the excitation signal and data acquisition and accurate and repeatable methods of positioning. Not without significance is also the development of miniature microphone transducers with a wide bandwidth, flat frequency response, and low noise. These facts prompted the authors to undertake the presented research. Some achievements in this field have already been presented in [15,17,18].

## 4. 2D Mechatronic Sound Intensity Probe Construction

**Figure 1:**
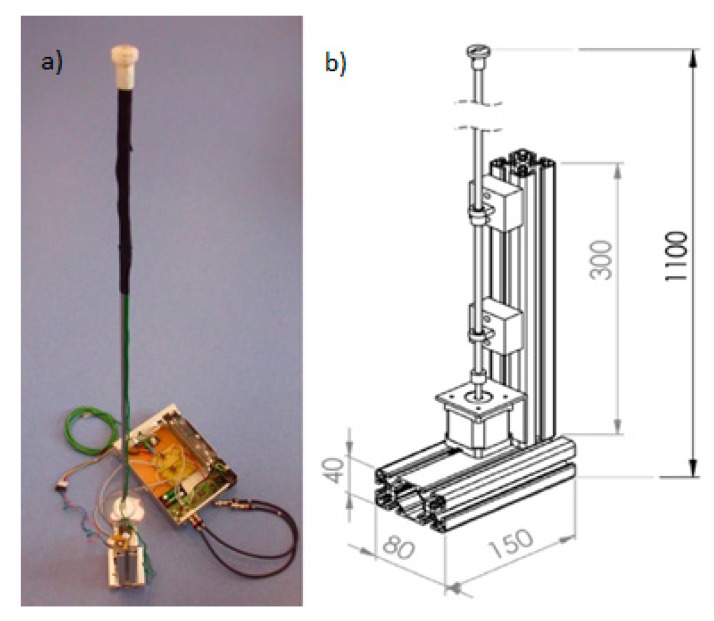
Construction of the 2D sound intensity probe based on two microphones (dimensional units are millimeters); (**a**) photo of real probe; (**b**) construction details.

**Figure 2:**
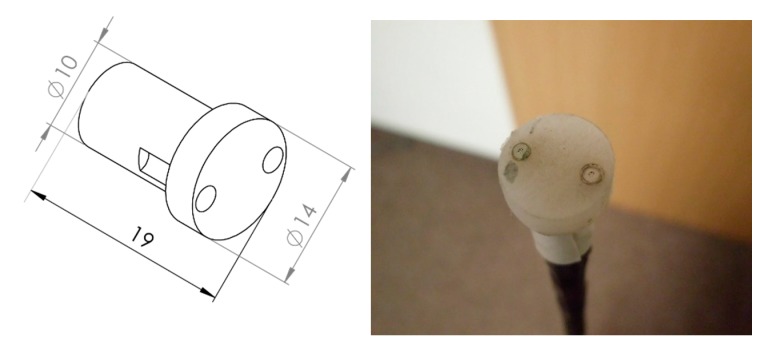
Construction details of the header of the mechatronic 2D sound intensity probe based on two microphones.

The measurements described in the next chapters were carried out with the use of the author’s own construction of the 2D probe. Although the probe has two microphones, it can also be used as a single-microphone probe. In this case, one microphone is switched off. Construction details are shown in Figure 1a,b. The probe consists of a base to which a stepper motor is attached. The motor shaft is connected to the bearing metal rod on which the plastic header with pair a of microphones is placed. Two cylindrical miniature electret microphones with a diameter of 1/10 inch (Sonion 8011 [19]) are placed in the header rolled out of ABS plastic. Details of the header are shown in Figure 2. The distance between the microphone axes is 10 mm. The microphones’ fronts are placed on the surface of the probe header. By placing the microphones perpendicular to the sound intensity measurement plane, the amplitude-phase characteristics are guaranteed to remain unchanged, especially in the high frequencies, as a function of the angular position of the microphone in relation to the sound source. The electronic part of the probe includes low-noise microphone preamplifiers with a battery power supply and a stepper motor controller with mains power supply. The whole measurement is performed by a dedicated LabVIEW virtual device responsible for the generation and acquisition of measurement signals and the generation of control sequences for the stepper motor. Probe positioning is carried out digitally in an open feedback loop.

## 5. Measurement of the Flow of Acoustic Energy in an Anechoic Chamber

In order to compare the metrological quality of the proposed measurement methods, two experiments were carried out at the West Pomeranian University of Technology in Szczecin, Poland in the hemi-anechoic chamber with a working space of 2 m (length) × 2.5 m (height) × 1.5 m (width). It can be assumed (it is a consequence of the hemi-anechoic chamber dimensions) that the conditions of the free field are maintained for frequencies above 300 Hz.

**Figure 3:**
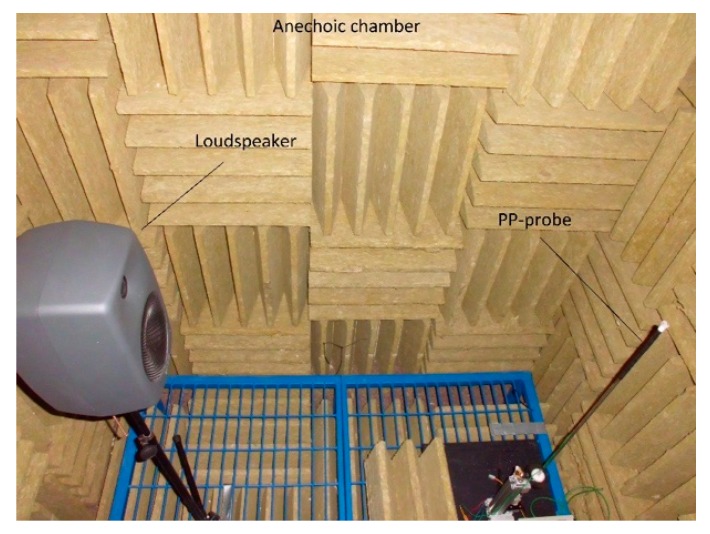

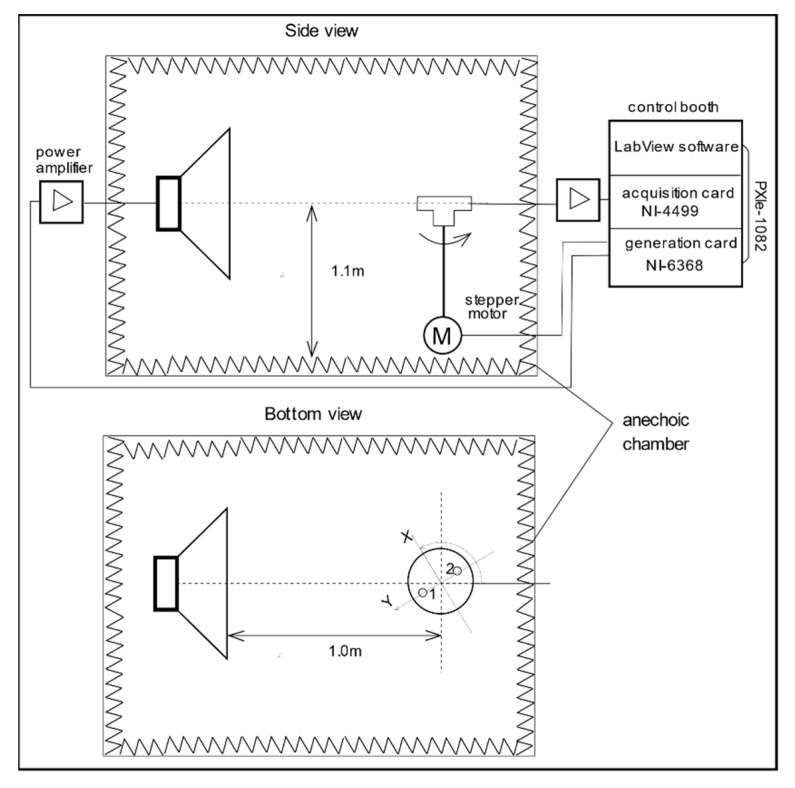
Experimental measuring workplace.

**Figure 4:**
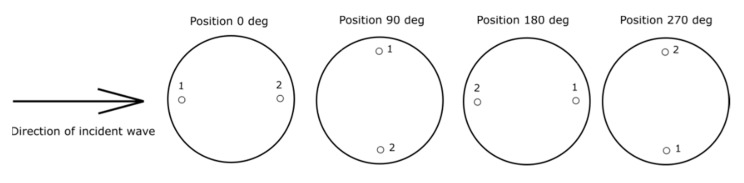
Positioning of the head with reference to the sound source.

The sound source was the Genelec 8040 [20] two-way active loudspeaker system. The measurement probe was located 1 m from the front of the loudspeaker. The location of the measuring station elements is shown in Figure 3. The application controlling the measurement process was launched on an industrial computer PXie-1082 equipped with acquisition and generation cards type: PXIe-6368 and PXIe-4499 [21]. The resolution of the analog-to-digital converter used is 24 bits, while the digital-to-analog converter is 16 bits. The data sampling frequency in all experiments was 100 kHz. The system ensured the synchronization of acquisition and generation processes with the accuracy of the 0.01 sample. The sensitivity of the whole acquisition path (microphone with a preamplifier) was 0.361 V/Pa for one microphone and 0.374 V/Pa for the second. The conventional axis of the probe lay on a straight line passing through the center of the microphones marked with “1” and “2” (Figure 4). During all measurements, the signals from both microphones were recorded simultaneously. To determine two components of sound intensity vector using a two-microphone probe, there is necessary to set the probe in two positions (e.g., 0 and 180 or 90 and 270). In case when the one-microphone method is used, four positions of probe are necessary to set. Of course, in this situation only one microphone is active.

The aim of the first experiment was to estimate the statistical error of measuring the component of sound intensity using the single-microphone method in comparison to the two microphones method using miniature electret microphones. In this experiment, the axis of the probe was set according to the axis passing through the geometric center of the loudspeaker set. In this position, tones of frequencies 250, 1000, and 4000 Hz were emitted and recorded by microphones in the measuring probe. The same measurement was made after turning the probe by 180⁰ (changing the position of microphones 1 and 2). In each of these positions, the measurement was repeated 100 times. By using a sinusoidal signal as an excitation, the number of necessary measurement data processing operations was reduced, and the final statistics of the obtained results show directly the quality of the measurement system and not the whole process of measurement data processing.

The second experiment consisted of examining the dependence of the obtained resultant value of the sound intensity vector on the angle of the acoustic energy flows through the probe. For this purpose, the probe head was rotated around its axis by a stepper motor. One step was equal to 1.8 deg. During full rotation of the head, pressure measurement from both microphones was recorded for 200 positions. Figure 4 shows four example positions of the head with microphones in relation to the direction of the flow of acoustic energy.

In each of these positions, the loudspeaker system emitted test signals in the form of test tones at frequencies as before and a logarithmically tuned sweep signal between 20 Hz and 20 kHz [22]. This allowed us to examine the properties of the measurement method as a function of frequency. The recorded values of voltage signals corresponding to the values of sound pressure at each point of measurement were recorded in the form of an LVM format. The measurement data were then processed using original scripts in the MATLAB software.

## 6. The Results of the Preliminary Experiment

This part of the article presents the results of the experiment described in the previous paragraph. The values of sound intensity are presented, which on the basis of measured values of acoustic pressures were determined using the formulas below:

(9) $$I\left(n\right)=u\left(n\right)\frac{p_{1}\left(n\right)+p_{2}\left(n\right)}{2}$$

(10) $$u\left(n\right)=\frac{1}{\rho f_{s}d}\overset{n}{\underset{k=1}{\sum }}p_{1}\left(k\right)-p_{2}\left(k\right)$$

where: *f_s_*—sampling frequency; *d*—distance between microphones; *ρ*—air density (1.2 kg/m^3^); *p*_1_, *p*_2_—instantaneous values of sound pressure registered by microphones in positions 1 and 2 (Figure 4); *n*—number of sample.

**Figure 5:**
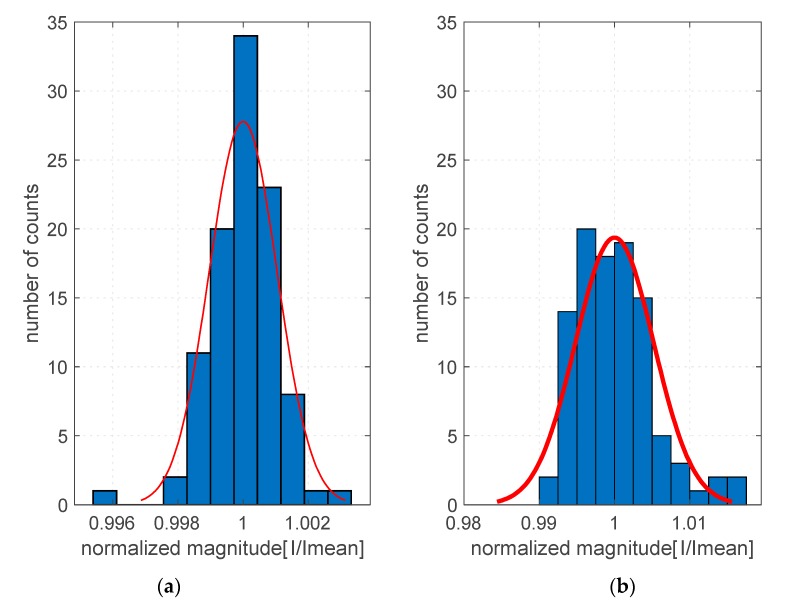
Statistical distribution of measurement results as a measure of repeatability of presented measurement methods. (**a**) Two-microphone probe and (**b**) one-microphone probe.

Figure 5 shows the histograms for the determined sound intensity values for both methods, and the normal distributions fitted to the data.

In the aim of statistical analysis of the collected data, the values of the active sound intensity were calculated as the average value of the intensity of the whole measurement time (Equation (8)).

Statistical parameters for the method using one and two microphones were calculated separately. The results are presented in Table 1.

**Figure 6:**
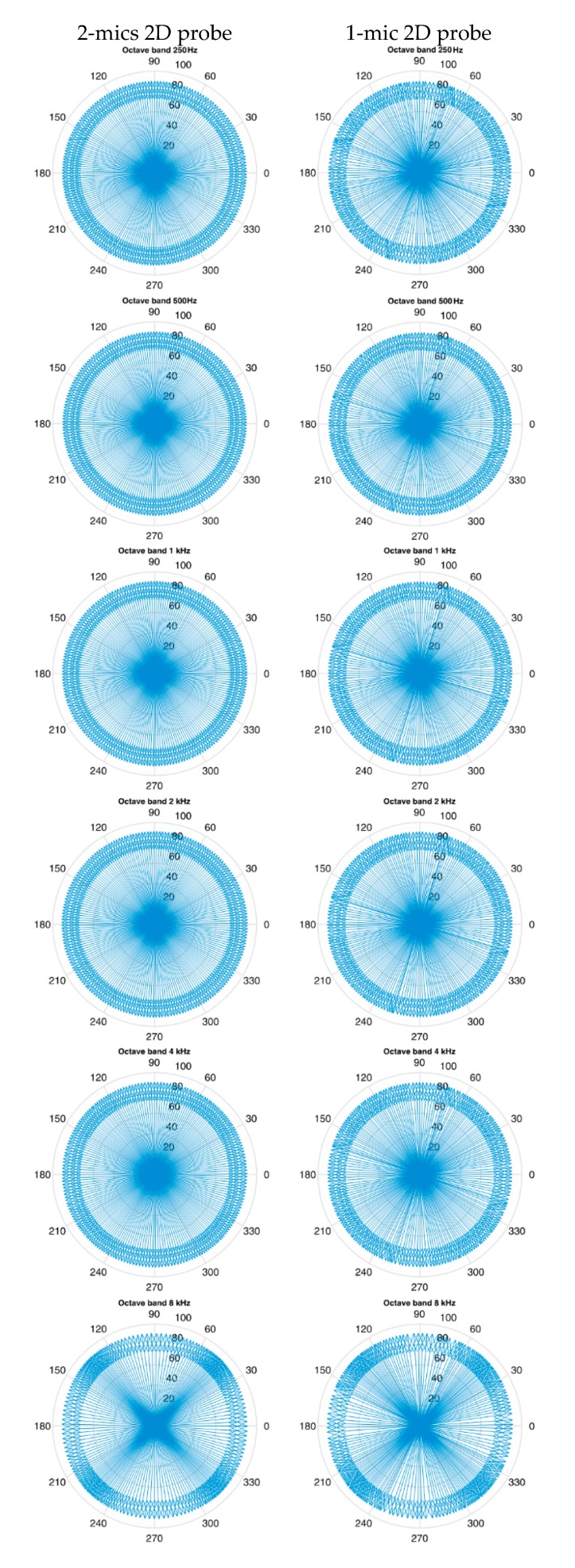
The distribution of the intensity vectors of the sound when the angular position of the probe relative to the sound source is incrementally changed for the octave bands from 250 Hz to 8 kHz.

Figure 6 shows the distribution of the intensity vectors recorded during the probe’s incremental angular position change with reference to the sound source. Column (a) shows the results for a two-microphone probe and column (b) shows the results for a single-microphone probe. The following lines present characteristic rosettes determined for subsequent octave bands in the range from 250 Hz to 8 kHz. Such imaging of the results allows to better recognize the qualitative differences between the tested methods.

**Figure 7:**
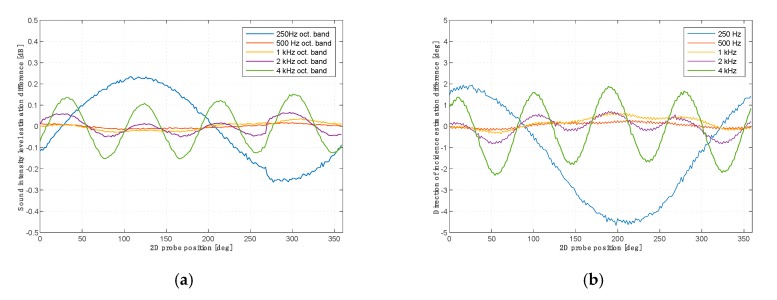
Variations of sound intensity magnitude (**a**) and deviation of the indicated angle from the nominal value (**b**) for the two-microphone probe for the subsequent angular positions of the probe.

The ideal intensity probe should record the sound pressure level and the spatial orientation of the vector independently of its setting relative to the sound source. The vectors shown in Figure 6 should be constant in length and their angle should correspond to the angular position of the probe. Due to measurement errors, this is not the case. Figure 7 shows the quantitative changes in the recorded sound pressure level and the deviation of the indicated angle from the nominal value for a two-microphone probe as a function of the angular position.

**Figure 8:**
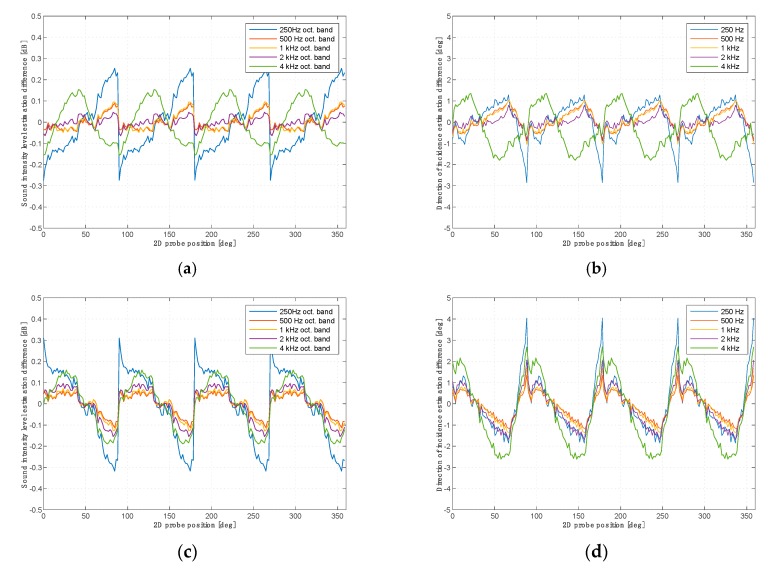
Variations of sound intensity magnitude (**a**,**c**) and deviation of the indicated angle from the nominal value (**b**,**d**) for a single-microphone probe for subsequent angular positions of the probe. Microphone 1 (**a**,**b**); microphone 2 (**c**,**d**).

Figure 8 shows the same dependencies for a single-microphone probe when using microphones 1 and 2.

## 7. Measurement of the Room Impulse Response

To show the utility of the proposed simplified methods of sound intensity measurement, an experiment was conducted to measure the impulse response of the room using sound intensity.

The impulse response obtained from the measurement procedure described in the international standard ISO 3382-1 [23] can be used to determine the reverberation time of a room. In the case of the impulse response obtained using the method described above, it is planned to use it for the spatialization of sound recordings, which is not the subject of ISO 3382-1. Therefore, not all solutions used in the measurement process must comply with it.

An omnidirectional sound source should be used to measure the impulse response. ISO 3382-1 describes in detail the parameters to be satisfied for a sound source to be considered omnidirectional. The most common source with parameters close to omnidirectional is the dodecahedron speaker. In work [24] can be found a description of other sources that can be used as alternatives to a dodecahedron speaker. The authors also mention the popular electrodynamic loudspeaker which, although it does not meet the requirements of the standard for omnidirectionality, gives sufficient results in numerous acoustic measurements. It is especially important that the electrodynamic loudspeaker is able to generate many times an acoustic field of identical parameters, which enables the use of averaging and increasing the signal-to-noise ratio—SNR (as opposed to, e.g., a gun or a pierced balloon). This transmitter is also characterized by its low price, which is particularly important for measuring systems that are to become more popular. In some measurements, such as speech intelligibility and distortion, a directional sound source is better than an omnidirectional sound source, as stated in the IEC 60268-16 international standard.

Another important aspect of measuring the impulse response is the character of the excitation signal that powers the sound source. The most popular types of signals are: rectangular pulses approximating Dirac delta, sinusoidal signal, pseudo-random sequences, and a swept-sine technique [22] proposed by Farina in 2000. ISO 3382-1 specifies that the signal level to background noise should not be less than 45 dB. However, when measurements are made using MLS (Maximum Length Sequence) signals, these requirements are lower because a better signal-to-noise ratio can be achieved by averaging. The swept-sine method provides a 20 dB SNR improvement over MLS methods.

**Figure 9:**
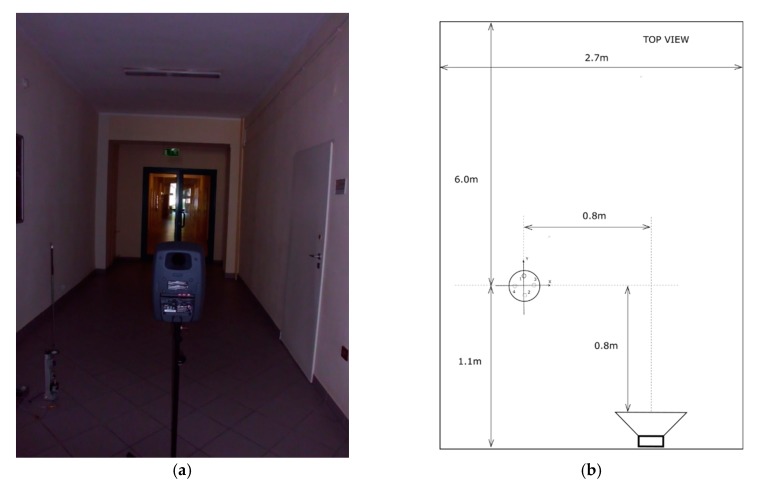

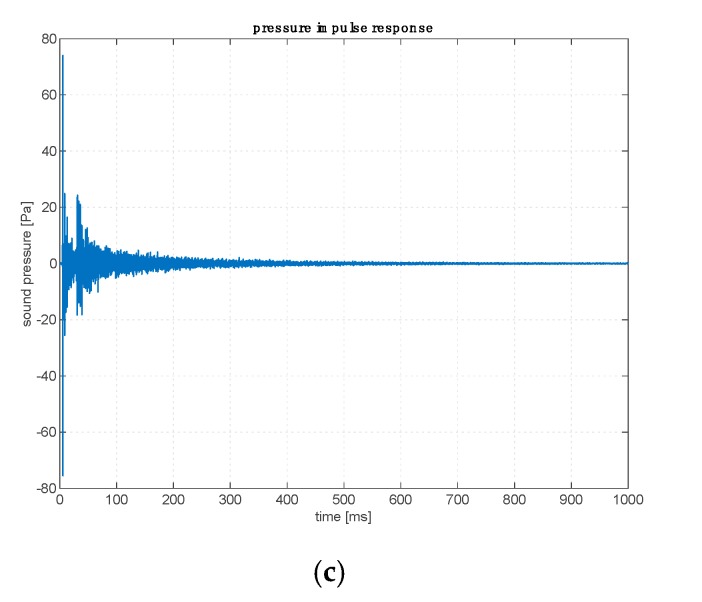
Conditions for measuring and the pressure impulse response of a sample room; (**a**) view of sample room; (**b**) dimensions of measurement setup; (**c**) standard pressure impulse response of the room.

Based on the above analyses, a Genelec 8040 speaker set was used as a source of sound in the experiment. It was powered by the signal of an exponential sweep tuned in the range of 20 Hz–20 kHz. Its duration was 10 s. Parameters of data acquisition devices remain the same as in the previous experiment. Figure 9 shows the view of the examined room and the location of the measurement probe and loudspeaker set and its standard pressure impulse response.

**Figure 10:**
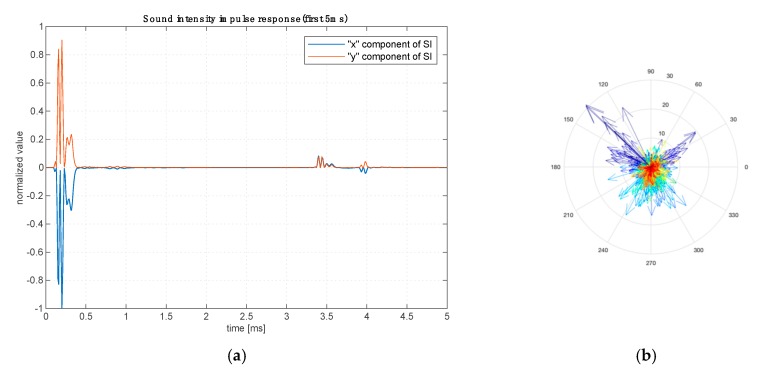
Sound intensity impulse response of a real room. Time evolution of two intensity vector components (first 5 ms) (**a**) and color-coded time evolution of sound intensity vector representation on a plane (whole response) (**b**).

Figure 10a shows the first 5 ms fragment of the obtained sound intensity impulse responses of the examined room for both components of the vector. We can see the direct sound impulse and first early reflections. Figure 10b shows the time evolution of the successive portions of energy reaching the probe using a color scale for the whole response. The dark blue color indicates direct sound and first reflection and finally the red color indicates the last reverberation tail.

This way of visualizing the impulse response of a room compared to traditional pressure responses allows for a time-spatial analysis of the propagation of acoustic energy in the room. The information about the amplitude, direction, and time dependencies of the incoming wave enables the detection of architectural elements in the room generating favorable or unfavorable reflections. Figure 10b shows the impulse response of the room in the polar diagram and in the logarithmic scale. Each arrow represents a bit of acoustic energy coming from one direction until energy from another direction starts to arrive to the probe.

**Figure 11:**
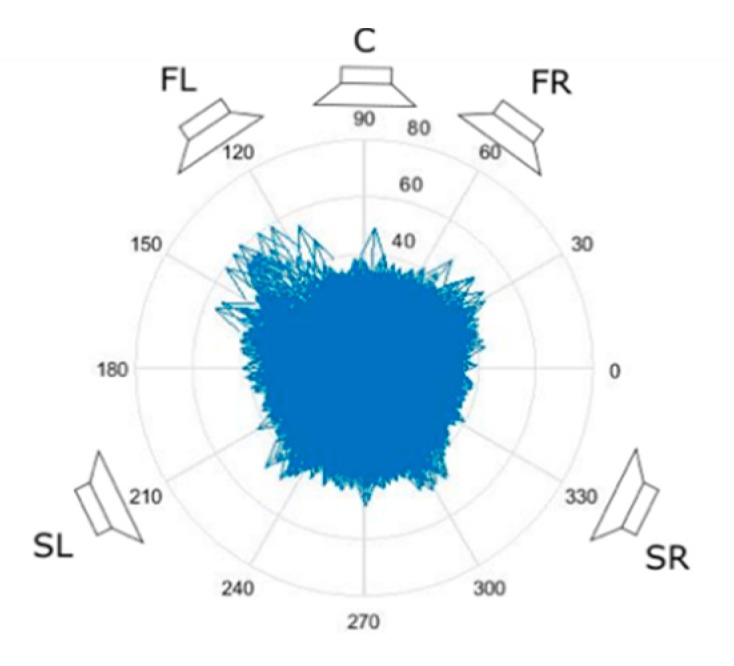
Sound intensity impulse response of a real room as source of spatial impulse responses for surround sound systems. (FL—front left; FR—front right; C—center; SL—surround left; SR—surround right).

A directional impulse response can also be used in sound spatialization systems by distributing it over the direction of energy delivery to elementary impulse responses that can be used in convolution processors to generate signals that supply individual channels of surround sound systems, as shown in Figure 11.

## 8. Discussion

The main novelty of the proposed single-microphone method is to replace the multi-sensor simultaneous measurement of pressure gradient components by a sequential single-sensor measurement. The technical problem of making at least two sensors (including measuring channels) with identical characteristics is replaced by the problem of repeating the measurement with identical excitation and identical condition of the measured object. The identity of excitation can be affected by the quality of the generating equipment and not perfect synchronization between generation and acquisition process in the following steps of the measurement procedure. At the current level of development of electronics and analog-to-digital and digital-to-analog converters built as single integrated circuits is the so-called codecs: this problem is mainly related to the stability of the clock signal, which cycles the converter. The clock jitter at the picosecond level seems to guarantee a negligible influence of this type of problem on the described method. However, it is difficult to unequivocally answer the question about the stationarity of the measured object. The element that certainly changes is the position of the transducer, which, in principle, is variable. To minimize the effect of this fact on the distribution of the measured field, the proposed solution uses a disk with an axis of symmetry passing through the axis of rotation and two microphones—in the single-microphone method, one transducer is active (measuring), and the other is used to minimize differences in the probe geometry when changing the position of the measuring microphone.

Equivalence of results can only be guaranteed if the subsequent steps of the measurement procedure are carried out under identical conditions. What is certainly not identical in subsequent measurements are the interference signals (noise) of the environment and the measuring equipment itself. In order to minimize the impact of these phenomena, it is necessary to ensure a sufficiently high ratio of the signal level measured to the noise level. Therefore, in the presented experiments, the measurements were carried out in an isolated room with a background level not exceeding 35 dBA and an SPL level of the signal measured at about 90 dBA. Thus, the results presented in Figure 5 can be interpreted as a limitation of the accuracy of the method resulting from the natural noise of the apparatus. The experiments showed that the assumption of the stationary character of the tested object, the conditions of measurement and the process of generation and registration of measurement data can be fulfilled with high accuracy, and the statistical error introduced is very small in comparison with other errors of the presented methods.

When measuring the intensity of sound for the purpose of determining the directions from which acoustic energy reaches the sensor, the main problem is to determine the directional characteristics of the 3D intensity probe. The ideal probe shall have the same accuracy in determining the direction of the energy flow independently of the location of the source in relation to the probe. Due to the lack of ideal geometric symmetry of the probe and the effects of diffraction of the acoustic field on the sensor, depending on the angle of attack of the acoustic energy flow on the microphone membrane, the accuracy of determination of the direction may vary. The second experiment showed that measurement errors in determining the magnitude and direction of the sound intensity vector are functions of the angle of attack of a sound wave. Comparing images and graphs for both probes, it can be stated that the two-microphone probe behaves differently from the single-microphone probe, although the maximum deviations are very similar and amount to ±0.3 dB and ±4 deg. It is worth noting that although the two-microphone probe gives less noisy results, its errors for different frequency bands have different cycles of repetition. The errors of a single-microphone probe change cyclically every 90 degrees at all frequencies. This is important from the point of view of the correct reproduction of the amplitude of the impulse responses.

## 9. Conclusions

Measuring the sound intensity allows us to obtain more information about the acoustic field around sources than just pressure information. Time and space information is very valuable and can be used, for example, in sound engineering and architectural acoustics. The development of low-cost methods of intensity measurement with sufficient accuracy is the basis for the dissemination of intensity methods in various fields of technology. The structures of p–p probes presented in the article fit well into this trend of development of measurement sensors. The sound intensity values are determined on the basis of a theoretical model. The accuracy of the determination of the direction of sound intensity is about 4 deg. The range of intensity measurement is limited from above by the maximum SPL value of the microphones used and for Sonion 8011 microphones the value is 112 dB SIL (Sound Intensity Level) (0.158 W/m^2^). The minimum value is limited by the noise level of the measuring system, the distance between the microphones in the probe, and the frequency range to be measured. The total noise level of the system is mainly influenced by the internal noise of the microphones, which for the Sonion 8011 used is 26 dB SPL. At this value the sensitivity of the probe is limited from below at 80 Hz by 72 dB SIL and at 1 kHz by 44 dB SIL.

The proposed method requires from the user only a simple calibration of the measuring channels (particularly relevant for the two-microphone method). It is limited to determining their sensitivity using a standard electroacoustic calibrator with an appropriate coupler interface.

At this stage of research the probe seems to be useful both for the measurement of properties of sound sources in the free field and of rooms in the diffusion field.

In the future, we plan to use this method in two areas of application. The first one is the analysis of noise generated by electrical machines using various synchronization methods of data acquisition with the rotation phase of the machine’s shaft. The second application is the use of directional impulse responses of rooms for the spatialization of sound recordings using convolution method.

## Figures and Tables

**Table 1:** Statistical parameters for the two-microphone and one-microphone measurement methods.

	Standard Deviation σ	3σ	95.7% Confidence Interval (In Normal Distribution)
2-mic meth	0.001	0.003	±0.01 dB
1-mic meth	0.005	0.015	±0.07 dB
